# Nutritional Assessment Methodologies: Challenges and Opportunities for the Full Realization of the Right to Food and Nutrition

**DOI:** 10.3389/fnut.2019.00035

**Published:** 2019-04-05

**Authors:** Hernando Salcedo Fidalgo, Juan Carlos Morales

**Affiliations:** FIAN Colombia, Bogotá, Colombia

**Keywords:** nutrition, food, human right to food, anthropometrics, nutritional profiles, health policies, children and adolescents

## Abstract

The structural isolation of nutrition from the human right to food has resulted in the technicalization and medicalization of the meaning and practice of nutrition, including in the field of nutritional assessments, which has led to the construction of public policies that lack a holistic perspective with a rights-based approach. Two main categories of nutritional assessments have been anthropometric measurements and nutritional profiles evident in the WHO and PAHO proposals related to the nutrition of children. In this paper, we present a critical discussion on the production and uses of both instruments in the evaluation of the growth and development of children and in the generation of global recommendations in public health with the objective of proposing alternatives for the measurement of malnutrition in communities affected by violations of the human right to food and nutrition. Our approach focuses on the construction not only based on the calorie-energy needs of the human body but also on food as a social, cultural and political process. It thus becomes an invitation to rethink nutrition from the notion of right to food to the implementation of research from participatory action.

## The Problem: the Structural Isolation of Nutrition From the Human Right to Food

The historical and legal isolation of nutrition from the human right to food as reflected in United Nations human rights instruments and in global governance processes interferes with the full realization of this human right^1^. The separation of food from its nutritional component contributes to: (I) the separation of food production from consumption; (II) private sector engagement that places inordinate attention on food production; (III) the technicalization and medicalization of the meaning and practice of nutrition; (IV) the exclusive nutritional focus on the first 1,000 days of a person's life and on pregnant and lactating women; and (V) the resulting reduction of women's roles to that of mothers^1^. The challenges emanating from the conceptualization of nutrition as a technical field delinked from food and the struggles of social movements, such as food sovereignty, are particularly reflected in how anthropometric and nutritional profiles—the leading tools in the measurement of malnutrition in children—are constructed and applied.

## Theory of the History (1) of Life and Paleo-Nutrition^2^ (2)

The contemporary configuration of scientific fields strengthened by positive empirical knowledge has given rise to the body of knowledge that we will call “Metric Sciences^3^.” Without considering metric obsession as a way of evaluating biological processes, it seems necessary today, in light of so-called scientific knowledge, to construct indicators that account for the vital phenomena that we want to control, evaluate, and transform. To measure is sometimes to qualify, if you have the appropriate comparison parameters and the statistical confirmation evidence. This is the case of models for evaluating the living conditions of human societies, which are established based on anthropometric parameters and nutritional metrics. These models of evaluation^4^ are probably inspired by the theories of the evolution of the species, in particular, the Theory of the History of Life, and in archaeological evidence that allow us to propose well-founded fictions of what such an evolution for the species could have been in order to reach the levels of biological organization that we experience today. Based on these premises we will make a critical reading of the analytical—interpretative model that dictates that anthropometry and nutritional metrics can indicate the welfare state of the human society.

## “Growth at Adolescence”

James Tanner in 1955 (4) proposed for the first time in the History of Public Health, the direct relationship between synchronic phenomena that had to do with the History of the Life of the species: the somatic growth of the human body and the development of puberty. He introduced in a heuristic way, concepts that put a biological neuro-endocrinological model in relation with the conditions of nutritional and social “stress,” which ultimately determined the characteristics of growth, development, and reproduction (4). This look revolutionized the medical context of the time, and connect it with the advances of the theoretical biology of the evolution of the species, and with the epidemiological and sanitary prediction.

The pioneering works of Tanner initially led to project the parameters built in their tables to other populations to establish comparative references, extrapolating the starting conditions of their samples onto other population samples that did not correspond to the context of the reference. This problem of the local production of reference anthropometric tables has remained until today as one of the critical pillars of the use of arbitrary standards to evaluate diverse human populations and to achieve a correlation of their levels of living conditions. Tanner showed, however, that the conditions of life configured variables of influence on the pubertal growth and development of the species, and that nutritional conditions and quality of life could be measured thanks to the dynamics of tissue growth and neuroendocrine development.

## Nutritional and Energetic Models

The growth model that values the contribution of proteins in nutrition along with the necessary calories for the development of tissue expansion comes into conflict when articulated with a neurological and endocrine matrix and is critically exposed in the construction of parameters that they measure the nutritional level with one or the other parameter, without putting them in relation. The reductionism of nutritional profiles tends to describe only the energy needed, in terms of kilocalories, to allow the vital biological function, as if the body were reduced to the same terms of the steam engine. In an epistemic sense, this reductionism tends to extrapolate the condition of the transformation of thermal and caloric energy into mechanical energy that the steam engine deploys, assimilating the human body to a thermodynamic system that consumes as much energy as available energy. This is a perversion of the complexity of what Tanner wanted to show from a heuristic point of view.

## WHO's Parameters and Local Parameters for Nutritional Evaluation

Returning to some of these debates, the World Health Organization (WHO) discussed in 1993 the conditions for assessing the growth and development of girls and boys, concluding that the conditions of comparative work based on what has been done since the 70s, were not sufficient or adequate (5). In 1994, the General Assembly constituted the Multicenter Growth Reference Study (MGRS) that between 1997 and 2003 generated new curves for assessing the growth and development of children around the world (6). The WHO text reports that: “The MGRS combined a longitudinal follow-up from birth to 24 months and a cross-sectional survey of children aged 18 to 71. Primary growth data and related information were collected from 8440 healthy infants and young breastfed children of very diverse ethnic and cultural backgrounds (Brazil, Ghana, India, Norway, Oman, and the USA).”

The purpose of the study was then to manage to put in parallel the diverse biological conditions of infantile human populations, to determine forms of standardization of their ways of development and to generate structural parameters of nutritional evaluation and living conditions. The problem that arose then and that prevails in methodological and epistemic terms regards the starting point that should be considered to determine that a population has reached the maximum development of its genetic potential under ideal conditions. In other words, how to show that if a girl or boy of a certain age and context reaches development patterns under ideal determinant variables expresses the maximum potential of life exhibited in terms of his or her anthropometry? This metric dilemma thus raises the ethical dilemma of standardizing anthropometric parameters based on a pattern whose objectivity is not attainable. In addition, the dilemma is constructed from thinking that the development of the species in the life history of its individuals, is carried out as that of the economic models of western capitalist societies. Again, an operational model created by the human and the economic is extrapolated to translate it into a biological reality represented in the measurement of bodies and their development.

In the same sense, the Pan American Health Organization (PAHO) Nutrient Profile Model assumes^5^ that a general recommendation can be formulated, the synthesis of which can be based on the exclusive control of a group of nutrients (salt, sugar, saturated fats, and trans fats) with the objective of “preventing the consumption of unhealthy foods” (7). It is necessary at this level of the presentation to explain why the PAHO criterion can become an exclusionary, reductionist, and risky scheme in terms of the actions that the States can implement around the regulation of the food industry.

1. The criterion of choice of nutrients does not cover all the basic requirements of food.
  The model presents “the formulation of norms and regulations applicable to hypercaloric foods and non-alcoholic beverages of little nutritional value” [(8) p. 6]. This approach presents the urgency of acting against the epidemic of metabolic diseases caused by excessive consumption of sugars, but in its eagerness to cover this spectrum, it excludes the recommendation of broader criteria based on comprehensive nutritional perspectives.
2. The criterion characterizes food intake only as an energy calorie balance (thermodynamic model).

The model conceives the body as a steam engine, which works based on an absolute energy equation in terms of contributions and calorie consumption. This model does not allow understanding the metabolism of nutrients as a complex of organic functions with access and transformation pathways that are diversified, changed, and adapted according to situations (of health and the physical environment in which it is developed) and foods processed by cells. The notion of “unhealthy” is relative, and only contemplates the problem of the concentrations of this group of nutrients, excluding the additives and the forms of processing.

The category of healthy or unhealthy goes through a sieve in which the relativization of the terms as “small” does not determine the scientific precision that the document intends. Therefore, this notion of “small” or “not healthy” must be deconstructed in light of what is really considered as food and what is not. That is why we propose the category of foodstuff for those products that in their processing cease to be food, that is, they stop offering adequate nutrients to the conditions that preserve, promote, and maintain the health of people, it seems to us more relevant, clear and precise.

The nutrient profile of PAHO is then directed to control the foodstuffs whose nutrient composition, from the selected list, corresponds to products harmful to health, because their excessive concentrations, within the thermodynamic model, have effects on metabolism linked with obesity.

It is then possible to think of specific profiles for the regions and appropriate to the conditions of each country, where the conditions of other forms of sanitary damage are estimated through the processing of foodstuffs. This objective should point to the integral projects of adaptation of the conditions of feeding of the populations. This perspective then includes the conditions of production of agricultural inputs, environmental impact and the transformation and commercialization of seeds, cultural conditions, and culinary traditions and food production, economic, social, and gender roles.

The profile of nutrients and the nutritional profile of a country must be the result of a civil and social reflection on the guarantee of their human right to adequate food and nutrition. Only in this way, will-science, alimentation, and cultural traditions—which as human traditions are modifiable—converge under an integral and holistic proposal that is not only energy data expressed in kilocalories.

## Proposal for a Rights-Based Alternative to Existing Nutritional Assessment Methodologies

The contributions of James Tanner to the conceptualization of the evolutionary expression of the species in terms of its development as a stature revealed heuristic criteria that methodologically have not managed to convert the complexity of its proposal into a valid and coherent system of evaluation of the conditions of life of a human group through anthropometry. Much is missing in the aspiration to build a method that allows anthropometric indicators to be placed as standards for the assessment of the nutritional and living conditions of a sample of people in a specific place and under specific conditions of social life.

The models of universalization that WHO and PAHO have sought to formulate to determine the nutritional conditions of human groups in healthy terms have been reflected in recommendations in the form of profiles that are limited by their conception. These are models that extrapolate human creations to the forms of expression of life and the biology of the species.

If we speak in terms of the human right to adequate food and nutrition, we would have to propose a complex model of analysis of food conditions, which considers a holistic framework that links the environment, food production, forms of production of economic and political systems with the display of somatic parameters that are not only anthropometric. This means evaluating nutrition with an approach that does not concentrate merely on those somatic parameters or other related biological or biochemical indicators, but also considers whether that nutrition responds to a diet that also satisfies (“nourishes”) other needs: affective; social; sensory; evocative; a sense of belonging to a group or territory.

This demanding aspiration is also a road under construction from the perspective of social transformation that inspires the right to be adequately nourished to live fully. FIAN has begun to forge this path by engaging in the development of participatory action research methodologies that seek to find alternative parameters to anthropometry that enables affected communities to meaningfully engage in self-reflection to understand and try to change their nutritional situation in a collaborative and sovereign manner. In this sense, with the communities, reflections are proposed on the complex act of feeding, and how a certain way of eating determines what is adequate and inadequate in nutritional terms, which, as was said, is not limited solely to the satisfaction of biological requirements, but also of a cultural, social, and political level.

## Author Contributions

All authors listed have made a substantial, direct and intellectual contribution to the work, and approved it for publication. HS contributed by the way of his research and field experience in the area of nutrition in FIAN Colombia (a human rights organization working for the right to food and nutrition), and JM apported the experience in front of the international advocacy of the rights of food by the way of his action in the direction of FIAN Colombia.

### Conflict of Interest Statement

The authors declare that the research was conducted in the absence of any commercial or financial relationships that could be construed as a potential conflict of interest.

## Footnotes

^1^Bellows et al. (1).

^2^The context of theoretical production of this concept would be consulted in Lucio and Francesca (3).

^3^This category is ours.

^4^At the time, strongly driven by colonial imperatives seeking to understand racial disparities from nutrition In Vernon, J. *Hunger* (2011)*. Modern history*. Universitat de Valencia. 2011. Pp. 122-135.

^5^The presentation of this discussion has been presented in detail in Salcedo, H., “De la demonización alimentaria a los perfiles nutricionales: intereses cruzados de un régimen corporativo”, in a volumen of FIAN Colombia in press, 2018.
